# Leveraging the role of pharmacists in vaccine education and uptake among newcomers to Ontario

**DOI:** 10.1177/17151635251380866

**Published:** 2025-11-06

**Authors:** Rochelle Spears, Caitlin Ford, Madison Fullerton, Hui Di Wang, Brent E. Faught

**Affiliations:** Brock University, St. Catherines, Ontario; Praxus Health, Calgary, Alberta; Praxus Health, Calgary, Alberta; Brock University, St. Catherines, Ontario; Brock University, St. Catherines, Ontario

## Abstract

**Background:**

Canada’s newcomer population has grown substantially over the past several years and will continue to increase. Newcomers are known to have lower vaccination coverage compared with the general population as they face unique barriers to vaccine education and access. This contributes to increased risk of vaccine-preventable diseases among newcomers. Pharmacists are the most accessible health care provider in the community, placing them in an ideal position to assist newcomers with vaccine education, access, and uptake.

**Methods:**

Using a qualitative descriptive methodology, we sought to understand the perceptions, barriers, and facilitators pharmacists experience when providing vaccine services to newcomers. We completed semistructured interviews with 12 pharmacists who practice patient care in Ontario.

**Results:**

The following 3 major themes were uncovered: (1) pharmacists are accessible health care providers in the community who are willing and motivated to provide vaccine services to newcomers to Ontario; (2) pharmacists do not proactively engage in vaccine education, but they can capitalize on opportunities to provide vaccine education to newcomers by incorporating conversations into routine pharmacy services, and (3) educational materials can support pharmacists and newcomers by addressing barriers and facilitators pharmacists encounter when providing these services.

**Discussion:**

The main findings from our research study indicate that pharmacists have the potential to improve vaccine education, access, and uptake among newcomers. Educational materials can support pharmacists by addressing the barriers and facilitators they encounter when providing these services to the newcomer population.

**Conclusion:**

Empowering newcomers through pharmacist-led vaccination services can increase vaccine uptake and improve the overall health and well-being of this growing population. *Can Pharm J (Ott)* 2025;158:xx-xx.

**Knowledge into Practice**

- Newcomers to Canada face unique barriers to vaccination, and despite a willingness to make informed decisions, they often lack tailored services and resources. Pharmacists, as trusted and accessible health care providers, remain underutilized in proactively engaging and educating newcomers about vaccinations.
- A 2-pronged approach that incorporates evidence-informed educational tools is essential to raise awareness and enhance pharmacists’ capacity to provide culturally relevant vaccine services to newcomers.
- By leveraging their community presence, pharmacists are well-positioned to improve vaccine uptake among newcomers through targeted and informed interventions.

**Mise En Pratique Des Connaissances**

- Les nouveaux arrivants au Canada sont confrontés à des obstacles particuliers en matière de vaccination, et malgré leur volonté de prendre des décisions éclairées, ils manquent souvent de services et de ressources adaptés.
- Les pharmaciens, en tant que prestataires de soins de santé fiables et accessibles, demeurent sous-utilisés dans la sensibilisation et l’éducation proactives des nouveaux arrivants en matière de vaccination.
- Une approche à deux volets qui intègre des outils pédagogiques fondés sur des données probantes est essentielle pour accroître la sensibilisation et la capacité des pharmaciens à fournir des services de vaccination adaptés à la culture des nouveaux arrivants.
- En tirant parti de leur présence au sein de la communauté, les pharmaciens sont bien placés pour améliorer le taux de vaccination chez les nouveaux arrivants grâce à des interventions ciblées et éclairées.

## Introduction

### Low vaccination coverage among Canada’s newcomer population

Canada’s newcomer population includes refugees, immigrants, and foreign workers who migrated to Canada within the past 5 years.^1^ By 2026, it is expected that newcomers will make up 30% of Canada’s population.^2^ Although newcomers are settling across Canada, in 2022, almost half of newcomers (44%) resided in Ontario.^3^

This population has lower vaccination coverage compared with Canadian-born residents^1,2,4^ due to unique barriers to vaccinations they face once settled in Canada.^1,4^ Barriers include cultural norm differences, knowledge gaps surrounding vaccines and vaccine schedules, vaccine hesitancy, insufficient access to primary health care, language barriers when seeking vaccine information, lack of affordable and accessible transportation, and lack of vaccine record sharing between countries.^1,4^ Notably, newcomers possess the desire to make informed decisions regarding vaccines. However, without tailored services, newcomers lack the resources to make these decisions.^1,2^

### Health care providers can mitigate vaccine barriers for newcomers

A major barrier newcomers experience is the inability to access primary care providers.^1,4^ This can result in vaccine hesitancy due to misinformation obtained from family or community members.^1,2^ Therefore, it is essential for health care providers (HCPs) to be available to offer credible and trustworthy vaccine education and to enhance vaccine confidence.^1,4^

It is also important for HCPs to use multipronged communication strategies when providing vaccine education to newcomers, such as offering community-based education sessions, one-on-one discussions, and information packages using pictograms with minimal writing to mitigate language barriers.^5-7^ Behaviour change theories, such as the Health Belief Model, should guide communication as they highlight self-efficacy and can contribute to newcomers’ ability to make informed decisions about vaccinations.^2,8^ HCPs should deliver concise vaccine recommendations, outlining the purpose, benefits, and risks of vaccines.^2,9^

### Pharmacists can facilitate vaccine uptake among newcomers

Pharmacists are readily accessible health care providers in Canada.^10^ Many pharmacies are open during off-peak hours, providing convenient and accessible health care. Due to their accessibility, 1 study found that pharmacists are often the first health care provider to assist newcomers with their health needs.^6^ Furthermore, Canadians trust pharmacists’ advice on vaccines^11^ and feel a high degree of patient satisfaction following pharmacist-administered influenza vaccinations.^12^ A systematic review and meta-analysis found that pharmacists improved the overall uptake of vaccines by up to 51% compared with usual care.^13^

As reliable, trusted, and accessible health care professionals, pharmacists are well-positioned in the community to provide vaccine services to newcomers and can play a significant role in increasing vaccine uptake.^10,12,13^ However, research on the barriers and facilitators that pharmacists face while offering vaccination services to newcomers, as well as how pharmacists can assist newcomers in increasing vaccine uptake, is lacking.

Thus, the primary objectives of the study were to (1) understand pharmacists’ perceived roles in addressing vaccine education, access, and uptake among newcomers to Ontario; (2) understand the knowledge gaps, barriers, and enablers that pharmacists experience when addressing vaccine education, access, and uptake among newcomers to Ontario; and (3) develop educational tools that assist pharmacists in engaging newcomers in conversations about vaccines.

## Methods

### Study design

A qualitative descriptive method was used as the impetus for this research approach to identify, understand, and depict the perspectives and experiences of the participants.^14^ The study method included semistructured interviews conducted in the English language, followed by an inductive thematic analysis of the data to decipher the findings.

### Participant recruitment

Pharmacists registered as Part A with the Ontario College of Pharmacists—practicing pharmacists who provide patient care in Ontario^15^—were eligible to participate in the study. Convenience sampling was used to recruit 12 participants through the primary researcher’s pharmacy networks. There was no compensation provided to participate in this study.

### Data collection/interviews

Semistructured interview questions were organized within the following topics: introductory questions, current scope of practice, and the pharmacists’ role in providing vaccine services to newcomers. Semistructured interviews were chosen as the method of data collection as they permit subjects to share viewpoints and experiences in a more humane approach.^16^ Demographic information collected from each participant included area of pharmacy practice—community, hospital, or both—and vaccination and injection certification status. The interviews were 60 minutes in length and took place on Microsoft Teams (Microsoft, Redmond, WA, USA) between April 22 and May 2, 2024. Interviews were recorded and transcribed verbatim. Interviews were conducted until the researchers obtained enough information to sufficiently answer the research questions.^17^ This method of qualitative data collection is relevant for this study as it minimizes the relevance and prevalence within the research of prior or personal assumptions from the researcher, aiming to accurately and truthfully represent the experiences associated with our subjects.^18,19^

### Data analysis

The primary researcher independently reviewed the interview transcripts and inductively developed an initial list of codes. The codes represent important commonalities across the dataset. A final codebook containing 9 distinct codes was agreed upon by 2 researchers. The primary researcher independently coded the dataset. Thereafter, 2 researchers collaborated using a thematic content analysis framework to generate a streamlined list of themes and subthemes.^20^ The themes represent subsets of the coded data that contain common meanings and ideas that were synthesized to describe the connections between them.

### Ethics

This research project was approved by the Brock University Research Ethics Board. The participants reviewed a consent agreement form and expressed verbal consent to participate prior to each interview.

## Results

### Participant characteristics

Twelve pharmacists participated in this study. All participants were registered as Part A with the Ontario College of Pharmacists. Five pharmacists practiced in a hospital setting, 3 pharmacists practiced in a community setting, and 4 pharmacists practiced in both settings. Eight participants were certified to provide injections in Ontario.

### Interview themes

**Figure 1:**
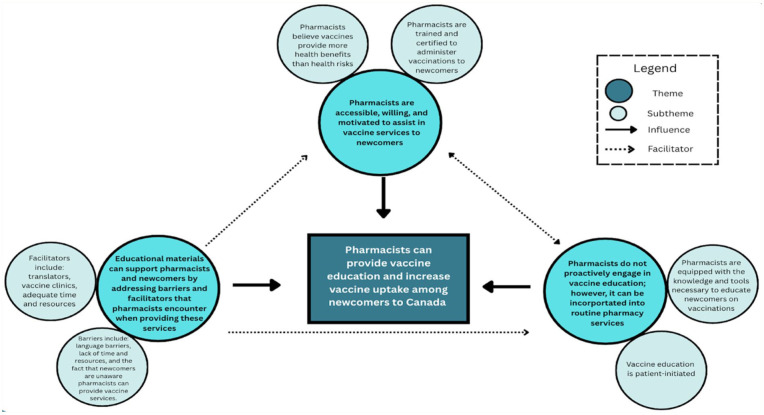
Theme schematic

Three major themes and 6 subthemes emerged surrounding the pharmacists’ experiences providing vaccine services for newcomers (Appendix Table A, available online under Supplementary Materials). While this study yielded 12 participants, saturation of themes and subthemes was attained as our analysis of qualitative data reached a point at which no new trends and/or information emerged from the data.^21^ Figure 1 was created to visually illustrate how the themes influence and facilitate one another.

#### Theme 1: Pharmacists are accessible health care providers in the community who are willing and motivated to provide vaccine services to newcomers to Ontario

All participants identified that community pharmacists are the most accessible health care provider to the public and are therefore in an ideal strategic position to provide vaccine services to newcomers. Participants conveyed that they are aware of the challenges in finding a primary care physician in Ontario, and if newcomers do not have a primary care physician, there is no one to provide them with reliable vaccine information.

“I think we play an integral role because we’re front-line, so we see people, whether they’re coming in for OTC [over-the-counter medications] or whether they’re getting their normal prescription drugs. So yeah, I mean we have a good strategic position.” (Participant 1)

Overall, pharmacists are willing and motivated to provide vaccine services to newcomers to increase vaccine uptake in this population, provided they have the time and resources.

“I’m just a firm believer in vaccinations in general because it’s a very proactive thing we can do to help prevent disease and reduce harm in society. So, I love vaccinating people, it’s just something I feel is really rewarding.” (Participant 10)

##### Subtheme 1a: Pharmacists believe vaccines provide more health benefits than health risks

All participants believe that vaccines provide more health benefits than health risks. Participants discussed the benefits that vaccines provide to individuals and communities through herd immunity and that these benefits far outweigh any risks associated with vaccines.

“Recognizing that they really make a difference, like the influenza vaccine truly reduces the rates of illness. And so, we have less patients coming into hospital with the disease, and if they do, they actually have shorter courses of the illness so because I see the benefits from [vaccinations], I’m highly motivated to actually advocate for it.” (Participant 9)

##### Subtheme 1b: Community pharmacists are trained and certified to administer vaccinations to newcomers

All participants identified that it is within pharmacists’ scope to administer vaccinations to individuals. Participants believe that community pharmacists are trained, certified, and comfortable administering vaccines to newcomers.

“[With] limited access to primary care providers, I think now more so than ever pharmacists are the front-facing accessible health care providers and can be for vaccines.” (Participant 8)

#### Theme 2: Pharmacists do not proactively engage in vaccine education; however, they can capitalize on opportunities to provide vaccine education to newcomers by incorporating conversations into routine pharmacy services

Participants discussed that they do not routinely initiate conversations about vaccine education with their patients. The reasoning was multifactorial; however, most cited time constraints, lack of resources, and workflow. Participants conveyed that they do provide vaccine education when asked by patients, and the frequency of vaccine education tends to be seasonal during the fall and winter months (e.g., respiratory virus season). Participants believe that incorporating vaccine education into pharmacists’ routine services, such as during one-on-one counselling, will assist with proactive vaccine conversations with newcomers.

“I usually never even start these conversations. It’s just on a practical sense, the workflow in community it’s so busy. . . . So either they ask questions like the friend got it, or someone got it and then they asked us about it, and then you talk a bit more, but usually there’s something that initiates it.” (Participant 7)

##### Subtheme 2a: Vaccine education is patient-initiated

Participants conveyed that pharmacists provide vaccine education only when they are asked by the patient.

“As far as actually having time to have one-on-one education, that tends to come in a way that’s driven by the patient. So, unless the patient calls or asks the question, it’s not usually a conversation that starts without the patient bringing it up. And I think that’s just a factor of time and availability.” (Participant 2)

##### Subtheme 2b: Pharmacists are equipped with the knowledge and tools necessary to advocate for and educate newcomers on vaccinations

Participants identified that pharmacists have the knowledge and tools necessary to provide vaccine education to newcomers. Participants discussed that pharmacists are comfortable providing education on the common vaccines (e.g., influenza, COVID-19, shingles, pneumococcal), and if they are unfamiliar with a vaccine, they have the resources to find the information necessary to educate newcomers.

“In my scope of practice there is definitely a role for [vaccine] education.” (Participant 4)

#### Theme 3: Educational materials can support pharmacists and newcomers by addressing barriers and facilitators pharmacists encounter when providing these services

Participants conveyed that educational materials tailored for newcomers would be beneficial as it would allow them to gain accurate vaccine information before interacting with HCPs. They felt that educational materials would inform newcomers that pharmacists are available to provide vaccine information. In addition, participants felt that educational materials would save pharmacists time by providing answers to questions newcomers may have prior to a discussion. Further, participants believe that educational materials for pharmacists could inform them of the barriers newcomers face surrounding vaccinations and provide them with resources to assist them with vaccine education.

“I think [educational materials] would be a really great idea. It would probably do the thinking for me right? Everything would be knowledge translated simplified, instead of me thinking off the top of my head how I would explain this for someone who may not be as familiar with this type of information.” (Participant 11)

“Everyone has busy lives . . . so somehow making it convenient for newcomers specifically, if we can set up these community hubs that would be great. And everything has to be well-advertised as well.” (Participant 12)

##### Subtheme 3a: Facilitators pharmacists experience when addressing vaccine services among newcomers include having a translator (family member) present, dedicated vaccine clinics, and adequate time and resources

Participants identified facilitators that assist them when providing vaccine services to newcomers, including having adequate time/resources to assist with vaccine services, group vaccine education sessions, and translators to help with communication.

“If there’s guidance in place, I feel like that would be more reassuring for both parties, the patient and the pharmacist.” (Participant 5)

##### Subtheme 3b: Barriers pharmacists experience when addressing vaccine services among newcomers include language barriers, lack of time and resources, and the fact that newcomers are unaware pharmacists can provide vaccine services

Participants identified barriers they experienced when providing vaccine services to newcomers, including language/communication barriers, and limited time and resources. In addition, participants discussed that there is a lack of knowledge in the community that pharmacists can provide vaccine services.

“The only reason would be lack of time . . . we’re so busy with so many other things. And then maybe if they have a lot of questions, then it’s hard to spend time with every patient to talk about vaccines when we have to do other duties like med histories and other counselling as well.” (Participant 3)

“The biggest barrier I can see in community pharmacies, we’re really relying on them to come to us. And so that’s the biggest barrier for any service we can provide to them.” (Participant 6)

## Discussion

The study results demonstrate that pharmacists are equipped with the knowledge and tools needed to educate newcomers regarding vaccines. This aligns with the results of a recent systematic review that found pharmacists shared a positive attitude toward their role as vaccine educators and administrators and were keen to expand this role.^22^ However, despite positive advancements in pharmacists’ scope of practice and their willingness to provide vaccine services, our study results indicate that pharmacists do not proactively engage in vaccine education with patients. These findings echo previous study findings that cite the reason pharmacists have a passive approach to vaccine education is due to the lack of time and resources required to proactively engage patients in conversations about vaccines.^23^ Thus, there is a need to support pharmacists in incorporating proactive vaccine education into their daily practice to increase vaccine uptake.

Considering the necessity of raising vaccination rates among Canada’s newcomer population, it is important to use pharmacists as vaccine resources in our health care system. Pharmacists are extensively distributed in the community, have convenient opening hours, cater to a walk-in policy, and are available to those without primary care physicians.^24^ For these reasons, pharmacists are in a prime position to address the vaccine accessibility barrier that newcomers experience. However, for this to occur, targeted resources must be available to encourage proactive vaccine conversations.

Many participants in our study expressed concern that newcomers may be unaware that pharmacists can provide vaccine services, which acts as a barrier to vaccine accessibility. Previous literature has also identified that a lack of public awareness surrounding the ability of pharmacists to provide vaccine services is one of the largest hurdles to any vaccination service.^25^ Furthermore, our study found that pharmacists believe vaccine education is driven by the patient, which is consistent with the literature that reports pharmacists take a passive approach to vaccine education.^23^ Together, these findings highlight 2 major gaps that exist in current pharmacy practice in Ontario: (1) newcomers will not seek out vaccine services from pharmacists if they are not aware that pharmacists can provide these services and (2) because pharmacists believe vaccine education is patient-driven, pharmacists will not proactively engage in these conversations. This contributes to missed opportunities in providing vaccine services to newcomers, accentuating the knowledge gap newcomers face when trying to access vaccine education.

A 2-pronged approach, using 2 distinct strategies, is necessary to address this gap and increase awareness among HCPs and patients.^26^ First, it is essential to raise awareness among the newcomer population that pharmacists are accessible vaccine educators, facilitators, and administrators. Promoting pharmacist-led vaccine services to the general population has led to an increase in the willingness to get the influenza vaccine.^25^ Raising awareness can be achieved by promotional materials targeted to newcomers that outline the importance of vaccines and highlight pharmacists as trusted and accessible HCPs. Second, it is necessary to support pharmacists by educating them on Ontario’s newcomer population and their unique vaccine barriers and by providing resources that can assist them with educating newcomers on vaccines.

To educate newcomers on the role that pharmacists play in vaccination, printed educational resources—such as health care infographics—can be used. Infographics that are designed for the patient populations present health care information through simple language and meaningful images in an attractive and easy-to-follow format.^27^ Infographics that are designed for HCPs provide an effective and efficient means of knowledge translation, as HCPs often have limited time to sift through and appraise the scientific literature.^22^

While resources may differ in their content and communication approach, they should address the perceived barriers identified by pharmacists in our study (Figure 1). For example, public-facing resources should address the key barriers newcomers experience in vaccination (Appendix Figure A), and HCP-facing resources should educate pharmacists on the unique vaccine barriers newcomers experience as stated in the literature (Appendix Figure B). To increase pharmacist engagement and motivation for practice change, pharmacists’ experiences, attitudes, beliefs, and perceived social norms should be incorporated.^22^

### Limitations

Several limitations were identified in our research study. First, only 1 researcher generated the codebook and coded the dataset. Second, sampling bias exists due to the results of convenience sampling. There also exist limitations in the generalizability of our results as all participants in the study practiced pharmacy within the Greater Toronto Area. Also, 4 of the 12 participants were not certified to administer vaccinations, and their experiences may not be generalizable to pharmacists who are certified to administer immunizations. Finally, it would be important to consider the view of newcomer populations in regards to pharmacist vaccination.

## Conclusion

Canada’s growing newcomer population is at increased risk of vaccine-preventable diseases because of their low immunization coverage. To prevent illness and death in this population, it is imperative to mitigate the vaccine barriers newcomers experience. Pharmacists are in an ideal position in the community to assist newcomers with vaccine education, access, and uptake due to their accessibility in the community, their knowledge, and their willingness to help. However, there is a need to address the perceived barriers pharmacists encounter when providing vaccine services to newcomers. Educational resources could address these barriers and assist pharmacists in providing vaccine services to newcomers while also enabling newcomers to make informed decisions about vaccines. By supporting pharmacists to empower newcomers with vaccine education and uptake, we can improve the overall health and well-being of this growing population. ■

## Supplemental Material

sj-pdf-1-cph-10.1177_17151635251380866 – Supplemental material for Leveraging the role of pharmacists in vaccine education and uptake among newcomers to Ontario Supplemental material, sj-pdf-1-cph-10.1177_17151635251380866 for Leveraging the role of pharmacists in vaccine education and uptake among newcomers to Ontario by Rochelle Spears, Caitlin Ford, Madison Fullerton, Hui Di Wang and Brent E. Faught in Canadian Pharmacists Journal / Revue des Pharmaciens du Canada
