# Supplementary material for: Leveraging the role of pharmacists in vaccine education and uptake among newcomers to Ontario
Source: Can Pharm J (Ott). 2025 Nov 6:17151635251380866. Online ahead of print. doi: 10.1177/17151635251380866 (PMC12592113; doi:10.1177/17151635251380866)
Supplement: sj-pdf-1-cph-10.1177_17151635251380866 – Supplemental material for Leveraging the role of pharmacists in vaccine education and uptake among newcomers to Ontario [file sj-pdf-1-cph-10.1177_17151635251380866.pdf]

APPENDIX

Table A. Themes and participant quotes

**Theme 1:** Pharmacists are accessible healthcare providers in the community who are willing and motivated to provide vaccine services to newcomers to Canada.	• "I think we play an integral role because we're frontline, so we see people, whether they're coming in for OTC or whether they're getting their normal prescription drugs. So yeah, I mean we have a good strategic position." (Participant 1)<br><br>• "I'm just a firm believer in vaccinations in general because it's a very proactive thing we can do to help prevent disease and reduce harm in society. So, I love vaccinating people, it's just something I feel is really rewarding." (Participant 10)<br><br>• "[With] limited access to primary care providers, I think now more so than ever pharmacists are the front facing accessible healthcare providers and can be for vaccines." (Participant 8)<br><br>• "In pharmacy school we were always told of that study that said that 95% of Canadians live within 5 kilometers of a pharmacy. And to put that into perspective, it means a lot of people have access to some sort of pharmacy in their area and I think even as a newcomer, if you don't have access to a primary care provider, you could easily stop into a pharmacy to ask questions that you have about common ailments. And I think that encounter is a really great opportunity for pharmacists to provide some education or to answer questions about vaccines." (Participant 11)<br>-

**Subtheme 1a:** Pharmacists believe vaccines provide more health benefits than health risks.	• "I believe that there's more benefits than risks and I guess in general that's like with herd immunity and everything. That's why we need all their routine childhood vaccines, and there's a lot of research that is done for vaccines. So, I think in general [vaccines] protect the individual and also the Community as well." (Participant 3)<br><br>• "Recognizing that they really make a difference, like the influenza vaccine truly reduces the rates of illness. And so, we have less patients coming into hospital with the disease, and if they do, they actually have shorter courses of the illness so because I see the benefits from [vaccinations], I'm highly motivated to actually advocate for it." (Participant 9)
**Subtheme 1b:** Community pharmacists are trained and certified to administer vaccinations to newcomers to Canada.	• "Most of [the vaccines] are intramuscular so they're all given the same way. If a subcutaneous one came up that that would be something that's not as common, but I think most of the Prevnar, Shingrix, influenza, COVID [vaccines] are all IM. So, I'm pretty comfortable with it." (Participant 1)<br><br>• "I mean [vaccines] that's our scope, and we need to be there to offer that service to patients (Participant 1).<br><br>• "I'm quite comfortable with [administering vaccines]." (Participant 4)<br><br>• "I think as pharmacists, [vaccine administration] is something that we can do. So instead of going to the family physician, pharmacists play a great role in terms of being able to administer vaccines." (Participant 10)

**Theme 2:** Pharmacists do not proactively engage in vaccine education; however, they can capitalize on opportunities to provide vaccine education to newcomers by incorporating conversations into routine pharmacy services.	• "I usually never even start these conversations. It's just on a practical sense, the workflow in community it's so busy […] So either they ask questions like the friend got it, or someone got it and then they asked us about it, and then you talk a bit more, but usually there's something that initiates it." (Participant 7)<br><br>• "I think um, making it easy and readily accessible or having information that is easy, simple to share and to provide them with, something to go home with would be a good starting point, because when you're getting these people at these other points of entry where they, you know, they know they need the health care system, so they're accessing it and then trying to identify what else we can offer them or what else we can share with them (Participant 2)<br><br>• "I think we could do a better job in terms of education perhaps. But unless you're doing a routine meds check and it becomes a category in the meds check, like immunizations that acts as a prompt for us to discuss it, I just don't see it being incorporated into the workflow of a pharmacist." (Participant 6).
**Subtheme 2a:** Vaccine education is patient-initiated.	• "As far as actually having time to have one on one education, that tends to come in a way that's driven by the patient. So, unless the patient calls or asks the question, it's not usually a conversation that starts without the patient bringing it up. And I think that's just a factor of time and availability." (Participant 2)<br><br>• "I don't do any meds checks in community, so I don't do any routine education on vaccines at that time either unless I'm asked." (Participant 6)<br>-

	• "If they're asking about [vaccines], we provide verbal information […], other than that, we're not really proactively looking to provide education. I think right now it's more them reaching out to us and then we're providing information as needed." (Participant 10)
**Subtheme 2b:** Pharmacists are equipped with the knowledge and tools necessary to advocate for and educate newcomers on vaccinations.	• "I think we definitely have the tools to educate and communicate." (Participant 1)<br><br>• "In my scope of practice there is definitely a role for [vaccine] education." (Participant 4)<br><br>• "I think most community pharmacists would [feel equipped…] especially the newer grads, I would say that have gone through vaccination programs as part of their curriculum in school." (Participant 6)<br><br>• "I think it would be good for pharmacists to educate, as [newcomers] don't have family doctors and so there's no one else doing it." (Participant 7)

**Theme 3:** Educational materials can support pharmacists and newcomers by addressing barriers and facilitators pharmacists encounter when providing these services.	• "Having information that is easy, simple to share and to provide them something to go home with would be a good starting point." (Participant 2)<br><br>• "If the resources can be used on social media or electronically that we can easily copy and paste and post up, that would be very helpful. I think we are really moving towards a digital stage even refugees and newcomers are looking online, Googling, so I think that would be very helpful where it's very easy for healthcare providers to post something up on their website. Saves everyone time." (Participant 7)<br><br>• "I think [educational materials] would be a really great idea. It would probably do the thinking for me right? Everything would be knowledge translated and simplified, and instead of me thinking off the top of my head how I would explain this for someone who may not be as familiar with this type of information." (Participant 11)<br><br>• Everyone has busy lives […] so somehow making it convenient for newcomers specifically, if we can set up these community hubs that would be great. And everything has to be well advertised as well." (Participant 12)

**Subtheme 3a:** Facilitators pharmacists experience when addressing vaccine services among newcomers include having a translator (family member) present, dedicated vaccine clinics and adequate time and resources.	• "If there is actual dedicated time, whether it's a group session […], a teaching session to help with the time barrier, as well if they have apps that they can use or maybe handouts in different languages that will help with the language barrier as well." (Participant 3)<br>• "If there's guidance in place, I feel like that would be more reassuring for both parties, the patient and the pharmacist" (Participant 5)<br>• "A majority of these individuals may not have ready access to a physician, but they certainly do to a pharmacist […] I still think that [pharmacists] are in a much better place than any other healthcare provider to [improve vaccine services to newcomers], if given the resources." (Participant 9)
**Subtheme 3b:** Barriers pharmacists experience when addressing vaccine services among newcomers include language barriers, lack of time and resources, and the fact that newcomers are unaware pharmacists can provide vaccine services.	• "The only reason would be lack of time […] we're so busy with so many other things. And then maybe if they have a lot of questions, then it's hard to spend time with every patient to talk about vaccines when we have to do other duties like med histories and other counseling as well." (Participant 3)<br>• "Language is the biggest hurdle. Written information would be helpful." (Participant 4)<br>• "The biggest barrier I can see in community pharmacies, we're really relying on them to come to us. And so that's the biggest barrier for any service we can provide to them." (Participant 6)<br>• "I don't know if many newcomers even know that pharmacists are able to provide [vaccine education]. I don't' think it's common knowledge. In fact, sometimes when I speak to the circle of friends that are not in the

	healthcare profession, they’re often surprised that pharmacists can do this.” (Participant 9)

Figure A. Infographic for newcomers

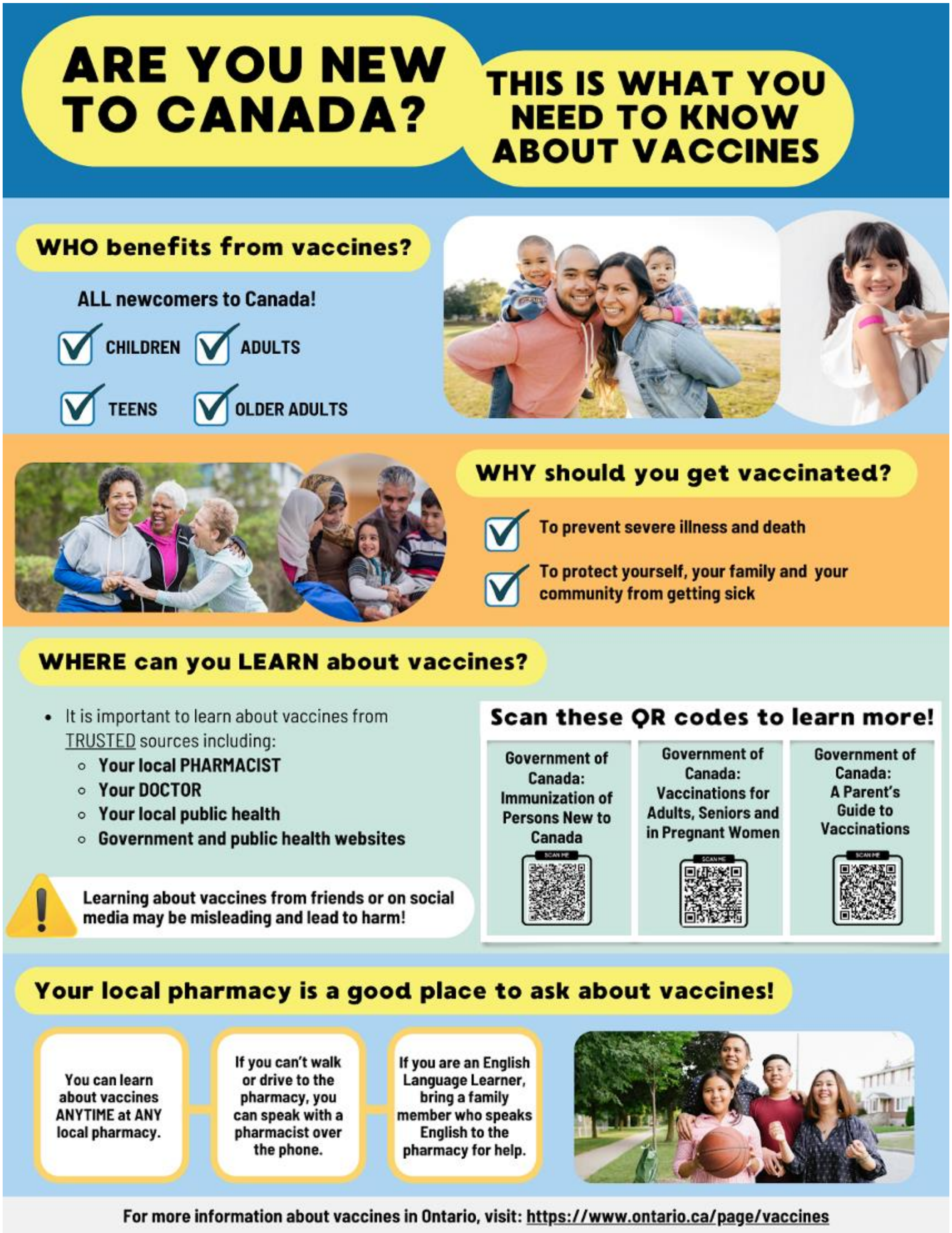

WHAT vaccines are recommended in Canada?

CHILDREN/TEENS

- MMR, DTP-Hib
- HepB, HPV
- Rotavirus, Varicella
- Pneumococcal
- Meningococcal
- Flu, COVID-19

ADULTS

- Flu
- COVID-19
- Routine vaccines if you missed any during your childhood

OLDER ADULTS

- Flu
- COVID-19
- Pneumococcal
- Shingles vaccine (Herpes Zoster)

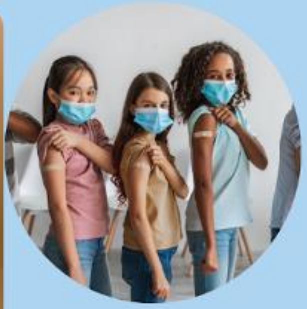

Do you have to PAY for vaccines?

Some vaccines are FREE

The government pays for public vaccines including:

- Routine childhood vaccines
- Some adult vaccines
- Flu vaccine, COVID-19 vaccine

Other vaccines may have a FEE

You may have to pay out of pocket for vaccines including:

- Travel vaccines
- Some adult vaccines

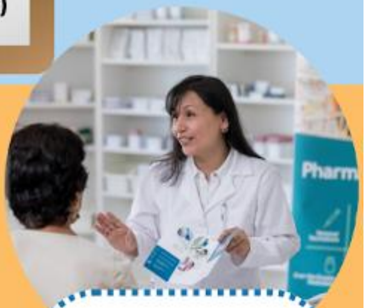

Speak with your pharmacist to find out more

WHERE can you get vaccinated?

Your Local Pharmacy

- Flu vaccine
- COVID-19 vaccine
- Adult vaccines* (e.g. shingles, pneumococcal, travel vaccines)

*certain vaccines may require a prescription from a doctor

Your Doctor's Office

- Routine childhood vaccines
- Adult vaccines (e.g. publicly funded vaccines, shingles, pneumococcal, travel vaccines)

Public Health Clinics

- Publicly funded vaccines (e.g., routine childhood and adult vaccines)

Scan to find a public health clinic near you

Check out these videos from the Public Health Agency of Canada!

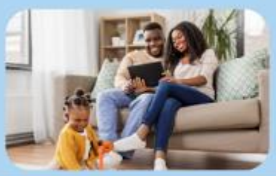

- Vaccine Safety and Effectiveness
- Diseases that Childhood Vaccinations Protect Against
- The Importance of the Childhood Vaccination Schedule
- Vaccination Side Effects Explained
- Lifelong Vaccination Journey

BE UP TO DATE WITH VACCINES!

TALK TO A PHARMACIST TODAY!

Updated in 2024

Figure B. Infographic for Pharmacists

A PHARMACIST'S GUIDE

Assisting Newcomers to Increase Vaccine Uptake

KEY POINTS

1. **Canada's newcomer population has grown substantially and continues to grow.**
2. **There is a need to increase vaccine uptake among newcomers to Canada.**
3. **Pharmacists are in an ideal position to facilitate vaccine uptake among newcomers.**

What should pharmacists know about Canada's Newcomer Population?

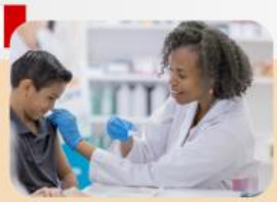

- Newcomers include **refugees, immigrants and migrant workers** who **migrated to Canada within the last five years**.[3]
- This population has **grown significantly** over the last several years, and it is expected to continue to grow as Canada welcomes ~500,000 newcomers annually.[4]

Barriers to Immunization Among Newcomers

Newcomers face unique barriers to vaccinations, including:

- Accessing primary care providers[3]
- Knowledge gaps [3 9]
- Language Barriers [3]
- Transportation barriers[3]
- Vaccine hesitancy[2]
- Cultural norm barriers[3]
- Childcare barriers[3]
- Vaccine Record Sharing[3]

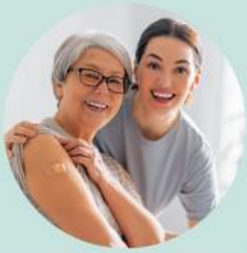

Pharmacists Can Facilitate Vaccine Uptake

Pharmacists can assist in increasing vaccine uptake among newcomers. Pharmacists:

- Are the most accessible healthcare provider.
- Are often the first healthcare provider to assist newcomers with their health care needs.[7]
- Have the knowledge and tools to advocate for and educate newcomers on vaccines.
- Have the skills to assist newcomers to navigate Canada's health system including vaccine access and provision.
- Are trained and certified to administer vaccines.

Are Newcomers to Canada eligible for publicly funded vaccines?

- **Immigrants and migrant workers** can apply for a government health insurance plan (e.g. Ontario Health Insurance Plan, OHIP) with their Canadian immigration status documents upon arrival. There is no waiting period.
- **Convention refugees and other protected persons** receive temporary coverage through the Interim Federal Health Program (IFHP) until their provincial health insurance plan is approved.
- The IFHP is funded by Immigration, Refugees and Citizenship Canada (IRCC), and administered by Medavie Blue Cross. It includes coverage for prescription medications and vaccines listed on provincial drug plan formularies.

What should pharmacists keep in mind?

- Newcomers may be **susceptible to vaccine preventable diseases** if their country lacked effective immunization programs.[6]
- It is **not mandatory for newcomers to be vaccinated before they enter Canada.** Some newcomers may be offered pre-departure vaccination services before they arrive in Canada, however these vaccines are voluntary.[5]
- **Immunization records may not exist or may be difficult to interpret** because of language barriers.[6]
- Immunizations schedules and vaccines may differ from those used in Canada. **Use your professional judgement** to assess the reliability of immunization records.

A PHARMACIST'S GUIDE

Assisting Newcomers to Increase Vaccine Uptake

How can pharmacists help Newcomers to Canada learn about vaccines?

- **Display educational materials** directed to newcomers in your pharmacy and on your pharmacy website.
- **Use clear, multi-pronged communication strategies** when educating newcomers on vaccinations, as this builds a trust in Canada's healthcare and increases vaccine uptake among newcomers[2] (eg. Infographics, government or public health agency websites, one-on-one discussions).
- Use **culturally responsive strategies when providing pharmacy services to build trust**[1]. This can be done by using a **patient-centered cross-cultural approach** when discussing vaccines with newcomers.
  - For example, ask 'How would a pharmacist help you with vaccines in the country you moved from?' This will empower newcomers and provide them with a sense of identity and autonomy[7].
- Use a **telephone interpreter service** for newcomers with a language barrier (**see Useful Resources for Pharmacists below*). If this is not feasible, **suggest newcomers bring a family member with them** for interpretation of vaccine education and services.
- Help newcomers find **Public Health Unit Vaccine Clinics** for publicly funded routine childhood and adult vaccines in their communities:
  Ontario's Ministry of Health - Public Health Unit Locator

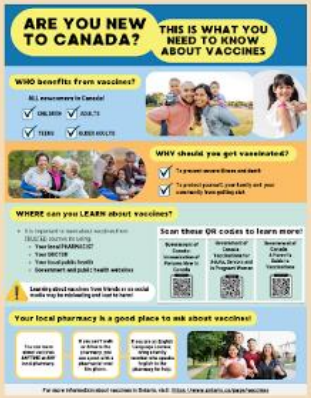

For more information on cultural competency, check out these online modules:

- Ontario Health Cultural Competence in Healthcare e-Learning Course
- Refugee and Global Health e-Learning Program

How can pharmacists help evaluate vaccination status for Newcomers to Canada?

- **Help newcomers identify the vaccines they were given** in their country of origin, and any **gaps in immunization** that may exist on their records.

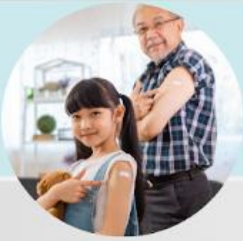

- For more information on evaluating immunization status of newcomers, see Government of Canada - **Immunization of Persons with Inadequate Immunization Records:** Canadian Immunization Guide.

Scan to access useful resources:

- The Canadian Immunization Guide
- Interim Federal Health Program Information
- IRCC Providers - Medavie Blue Cross Resources
- Settlement.Org - Newcomer settlement services
- Telephone Interpreter Services Access Alliance

Updated 2024

References:

1. Aghajafari, F., Ness, A., Wall, L., Weightman, A., Lake, D., Anupindi, K., Moorthi, G., Kuk, B., Santana, M., & Coakley, A. (2023, October 26). An Exploration of COVID-19 Vaccination Models for Refugees and Newcomer Immigrants in a Canadian City. Research Square. https://doi.org/10.21203/rs.3.rs-3471473/v1
2. Brooks, S. P., Sidhu, K., Cooper, E., Driedger, S. M., Gisenya, L., Kaur, G., Kniseley, M., & Jardine, C. G. (2024). The influence of health service interactions and local policies on vaccination decision-making in immigrant women: A multi-site Canadian qualitative study. Vaccine, 42(11), 2793–2800. https://doi.org/10.1016/j.vaccine.2024.03.014
3. Fullerton, M. M., Pateman, M., Hasan, H., Doucette, E. J., Cantarutti, S., Koyama, A., Weightman, A. M., Tang, T., Coakley, A., Currie, G., Fabreau, G., Constantinescu, C., Marshall, D., & Hu, J. (2023). Barriers experienced by families new to Alberta, Canada when accessing routine-childhood vaccinations. BMC Public Health, 23(1). https://doi.org/10.1186/s12889-023-16258-7
4. Government of Canada. (2022, November 1). Notice – Supplementary Information for the 2023-2025 Immigration Levels Plan. Www.canada.ca. https://www.canada.ca/en/immigration-refugees-citizenship/news/notices/supplementary-immigration-levels-2023-2025.html
5. Government of Canada; Immigration, R. and C. C. (2012, November 7). Do I have to get certain vaccinations before I arrive in Canada? Ircc.canada.ca. https://ircc.canada.ca/english/helpcentre/answer.asp?qnum=1346&top=33
6. Public Health Agency of Canada. (2015). Page 10: Canadian Immunization Guide: Part 3 – Vaccination of Specific Populations - Canada.ca. Canada.ca. https://www.canada.ca/en/public-health/services/publications/healthy-living/canadian-immunization-guide-part-3-vaccination-specific-populations/page-10-immunization-persons-new-canada.html
7. Ingar, N., Farrell, B., & Pottie, K. (2013). Building a welcoming community. Canadian Pharmacists Journal / Revue Des Pharmaciens Du Canada, 146(1), 21–25. https://doi.org/10.1177/1715163512472321
8. Sepp, K., Kukk, C., Cavaco, A., & Volmer, D. (2020). How involvement of community pharmacies improves accessibility to and awareness about flu vaccination? – An example from Estonia. Expert Review of Vaccines. https://doi.org/10.1080/14760584.2020.1825949
9. Wilson, L., Rubens-Augustson, T., Murphy, M., Jardine, C., Crowcroft, N., Hui, C., & Wilson, K. (2018). Barriers to immunization among newcomers: A systematic review. Vaccine, 36(8), 1055–1062. https://doi.org/10.1016/j.vaccine.2018.01.025

Spears R, Ford C, Fullerton M, et al. Leveraging the role of pharmacists in vaccine education and uptake among newcomers to Ontario. Can Pharm J (Ott) 2025;158. DOI 10.1177/17151635231380866.
